# First report of invasive *Aspergillus* rhinosinusitis in a critically ill COVID‐19 patient affected by acute myeloid leukemia, northeastern Iran

**DOI:** 10.1002/ccr3.4889

**Published:** 2021-10-04

**Authors:** Neginsadat Hosseinikargar, Reza Basiri, Mohammad Asadzadeh, Mohammad Javad Najafzadeh, Hossein Zarrinfar

**Affiliations:** ^1^ Department of Parasitology and Mycology School of Medicine Mashhad University of Medical Sciences Mashhad Iran; ^2^ Lung Diseases Research Center Mashhad University of Medical Sciences Mashhad Iran; ^3^ Department of Microbiology Faculty of Medicine Kuwait University Jabriya Kuwait; ^4^ Allergy Research Center Mashhad University of Medical Sciences Mashhad Iran

**Keywords:** AML, *Aspergillus*, COVID‐19, Iran, rhinosinusitis

## Abstract

This is a report of established invasive *Aspergillus* rhinosinusitis in a patient diagnosed with COVID‐19 and afflicted by AML, which was initially considered to be rhinocerebral mucormycosis.

## Graphical abstract

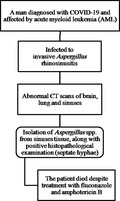

## 1 INTRODUCTION

A 38‐year‐old man diagnosed with COVID‐19 and affected by acute myeloid leukemia (AML) developed invasive *Aspergillus* rhinosinusitis. He presented dyspnea, blurred vision, and a headache. Invasive *Aspergillus* rhinosinusitis is rarely reported in COVID‐19 patients, and the diagnosis was obtained using a CT scan, histopathological findings, and mycological techniques.

Invasive aspergillosis (IA) is one of the most common opportunistic fungal diseases in susceptible patients.^1^, ^2^ IA is a major cause of mortality among patients with underlying immunodeficiencies, especially those with hematopoietic stem cell transplantation (HSCT), patients with hematological malignancies, and those admitted to intensive care unit (ICU).^3^, ^4^, ^5^, ^6^ IA can be seen in different clinical forms. However, infection severity can vary greatly, depending on the pathogen, the physiologic and immunologic condition of the host and any medical treatment received.^1^ IA rhinosinusitis is a rare and life‐threatening opportunistic infection that occurs predominantly in immunocompromised individuals and is caused by various *Aspergillus* species.^7^, ^8^ IA is rarely reported among the coronavirus disease of 2019 (COVID‐19).^9^ Recently, severe viral pulmonary infections have been shown to be associated with an increased risk for IA.^9^ However, most reports have been published on *Aspergillus* findings in COVID‐19 patients may be a proposition of a new disease entity, COVID‐19‐associated aspergillosis. Other reports of opportunistic fungal infections such as candidiasis and mucormycosis are increasing in COVID‐19 patients, so it is important to identify these fungal agents correctly before successful treatment.^8^, ^10^, ^11^ However, it is not yet known whether patients with severe COVID‐19 are at similar risk for IA development or not. From early reports from Wuhan, we know that patients with COVID‐19 may develop complicated fungal infections.^9^, ^10^ On the contrary, fungal disease related mortality in leukemia patients is a worrying matter.^4^ After the lungs, the sinuses are one of the most susceptible organs to opportunistic fungal infections, especially aspergillosis.^8^ In the study by Ahmed El‐Kholy et al., 36 patients with invasive fungal sinusitis affected to COVID‐19 showed a survival rate 63.89%; however, early management with antifungal therapy and surgical debridement recommended for better outcomes and higher survival.^12^

We describe a case of invasive *Aspergillus* rhinosinusitis in a critically ill COVID‐19 patient affected by acute myeloid leukemia (AML) from northeastern Iran.

## 2 CASE REPORT

**FIGURE 1:**
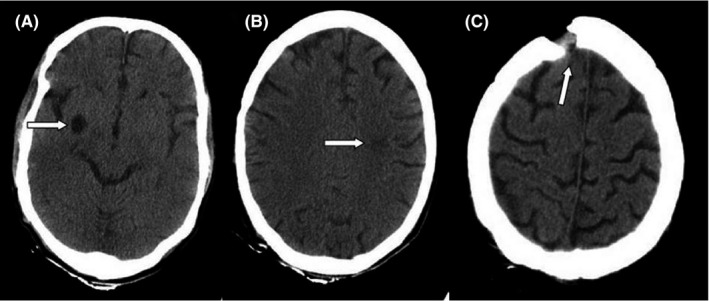
Computed tomography (CT) scans of the brain of the patient. A hypodensity in the left peritoneal (A), CSF‐like density in the right basal ganglia (B), a bone defect in the right frontal (C)

**FIGURE 2:**
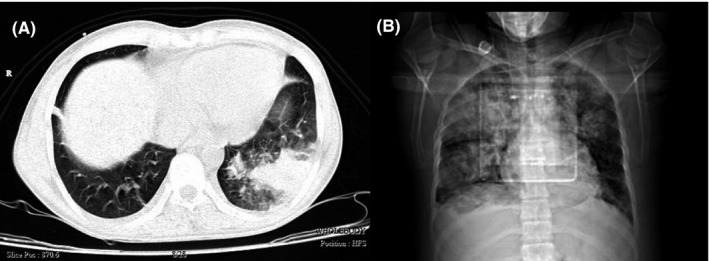
Computed tomography (CT) scans of the chest suggestive of COVID‐19. Ground‐glass opacities patch and consolidation patch in the peripheral areas of the lobes of the lungs (A), multiple consolidation and confluent lesions and organic pneumonia (B)

**FIGURE 3:**
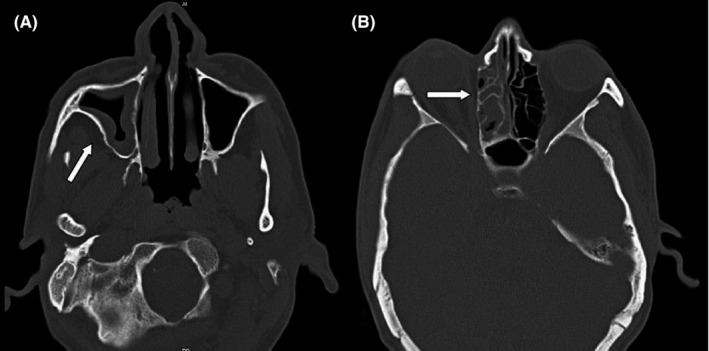
Computed tomography (CT) scans of the sinuses in Invasive *Aspergillus* rhinosinusitis. Thickness in the maxillary, sphenoid, and frontal sinuses (A), opacification in the right ethmoid sinus (B)

**FIGURE 4:**
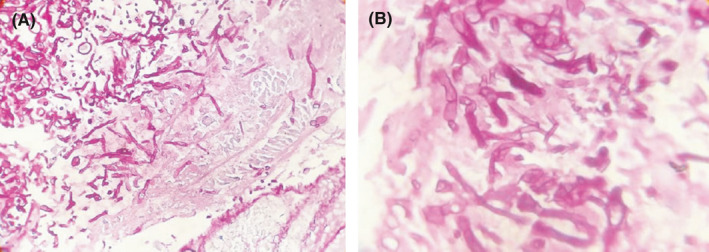
Histopathological examination using periodic acid–Schiff (PAS)—stained, dense accumulation of fungal hyphae with infiltration of surrounding necrotic tissue (magnification 400) (A), numerous septate hyphae with dichotomous branching (magnification 1000) (B)

A 38‐year‐old man living in Roshtkhar (Khorasan Razavi, northeastern Iran) was admitted to Ghaem Hospital in Mashhad in April 2021. He complained of blurred vision, shortness of breath, fever, cough, and tears in his right eye, and pain in his leg bone (tibia). In the past, he had undergone three courses of chemotherapy due to acute myeloid leukemia (AML) and had relapsed again. During hospitalization, his hematological findings showed white blood cell (WBC) = 154.3 × 10^3^/µl, red blood cell (RBC) = 2 × 10^6^/µl, hemoglobin (Hb) = 6.5 g/dl, hematocrit (Ht) = 18.5%, platelet (Plt) = 18 × 10^3^/µl, mean corpuscle (cell) volume (MCV) = 92.5 fl, mean corpuscular hemoglobin (MCH) = 32.5 pg, mean corpuscular hemoglobin concentration (MCHC)= 32.1 g/dl, red blood cell distribution width (RDW)‐CV = 14%, neutrophil (Neu) = 1%, lymphocyte (Lym) = 3%, blast cells 96%, and smudge cell (++). Moreover, other laboratory findings showed pco_2_ = 31 mmHg, Po_2_ = 57.7 mmHg, uric acid = 8.3 mg/dl, and also calcium, phosphorus, magnesium, albumin, urea, creatinine, sodium, potassium, SGOT, SGPT, bilirubin were in the normal range. Urine culture was negative and urine analyses showed no abnormal findings. Blood cultures were negative after 48 and 96 h too. Due to the low level of blood platelets, this patient underwent serum therapy, which despite receiving platelets, still had a decrease in platelets. In 2020, he underwent three surgeries due to fungal sinusitis. At the time of admission in April 2021, his COVID‐19 test result was positive, and he was diagnosed with AML. The following results were obtained during CT scans; in the brain CT scan (Figure 1), a hypodensity was seen in the left peritoneal, CSF‐like density in the right basal ganglia suggests an old infarction; a bone defect was seen in the right frontal, and no other significant abnormality were seen. In lung CT scan (Figure 2), grand opacity patch and consolidation patches in the peripheral areas of the lobes of the lungs, along with multiple consolidations and confluent acute respiratory distress syndrome (ARDS) lesions and organic pneumonia, were observed. Subsegmental atelectasis was seen in the superior lingula segment and the right middle lobe (RML) medial segment. The CT severity of lung involvement was score 20 (of total 24). Moreover, a mediastinal lymphadenopathy was seen in the carina. On paranasal sinuses (PNS) CT scan (Figure 3), an increase in mucosal thickness was observed in the maxillary, sphenoid, and frontal sinuses of the right and left ethmoid. Also, an opacification was seen in the right ethmoid sinus. Sinus debridement was performed via a cystoscopy, and secretions and tissue specimens were submitted to the medical mycology and pathology laboratories for further work‐up. Direct microscopic examination of clinical specimens in 15% potassium hydroxide (KOH) showed septate, branched, and hyaline hyphae. Moreover, the clinical specimen was inoculated on Sabouraud dextrose agar (SDA) with chloramphenicol and then incubated at 35℃ for 4–6 days. The culture result yielded a rapidly expanding hyaline filamentous fungal colony. Macroscopic (the round yellowish‐green colony) and microscopic examination (conidiophores and terminating vesicles giving rise to the phialides, the rounded conidia) revealed an *Aspergillus* spp. Unfortunately, the *Aspergillus* was contaminated and missed in our laboratory before species identification via a molecular method. Histopathological examination using periodic acid‐Schiff (PAS)—stained sections showed a dense accumulation of fungal hyphae that in some places there was a middle septum (septate), rarely lateral ruptures, infiltration of surrounding necrotic tissue, and vascular invasions (Figure 4). Fluconazole capsule (150 mg/day) and amphotericin B injection (50 mg/day) were prescribed to treat this patient. Moreover, he received various antibiotics such as cefepime, vancomycin, cefazoline, and meropenem. Eventually, he was hospitalized for 1 month, 3 weeks in the internal medicine ward, and 1 week in the ICU. Unfortunately, the patient died due to a cardiac arrest after 30 days of hospitalization.

## 3 DISCUSSION

IA is one of the most common opportunistic fungal infections among immune disorders and was recently reported in COVID‐19 patients.^9^ Different clinical forms of aspergillosis have been reported, which vary depending on the patient's condition. Although the pulmonary form is one of the most common and has a higher mortality rate, other clinical forms can be severe and fatal.^4^ It is unclear whether some infectious disease, such as COVID‐19, could be a predisposing factor for developing fungal infections. However, reports of fungal infections such as candidiasis, aspergillosis, and mucormycosis in patients with COVID‐19 are increasing, especially among critically ill patients with severe COVID‐19.^9^, ^10^, ^11^ The current case is the first of IA rhinosinusitis in an ICU patient with COVID‐19 and affected AML from northeastern Iran.

A 38‐year‐old man, who was diagnosed with COVID‐19 and affected to AML, developed invasive *Aspergillus* rhinosinusitis.

Although opportunistic hyaline fungi such as *Aspergillus*, *Penicillium*, and *Rhizomucor* are commonly reported causes of fungal rhinosinusitis, some coenocytic fungi such as *Bipolaris*, *Curvularia*, *Alternaria*, and *Neoscytalidium* are also have been observed with increasing frequency.^7^, ^8^, ^13^, ^14^ Generally, fungal rhinosinusitis is seen in warm and dry climatic conditions, and it is particularly common in young men from rural areas.^7^, ^8^, ^15^ In the current case, also this patient had rural communities. However, due to the increase in droughts in recent years in some areas and the consequent increase in dust, the disease can increase in other areas as well. In some areas, especially the Middle East, *A. flavus* is most commonly associated with both chronic invasive and granulomatous *Aspergillus* rhinosinusitis.^7^, ^8^ The precise identification of *Aspergillus* isolates can lead to successful treatment of patients.^1^ Unfortunately, in the present study, it was impossible to identify the cause of this infection at the species level due to the loss of the *Aspergillus* colony. In previous studies in this area, *A. flavus* was identified as the most common cause of fungal rhinosinusitis.^7^, ^8^ This could be due to the presence of most *A. flavus* spores in the air, which are affected by the climatic conditions of this region.^16^ In sinus involvement by fungal agents, most cases of maxillary sinuses show this involvement.^8^, ^14^ However, both the maxillary and ethmoid sinuses were impaired and thickened in the mucosa. However, the unintended effects for the patient may have been caused by the aforementioned intracranial vascular complication, leading to a severe ischemic insult. Because the conditioning regimen eradicates malignant cells, ineffective hematopoietic cells, and host immune cells among patients with hematological malignancies and HSCT, they are more sensitive to opportunistic fungal infections, particularly *Aspergillus* infections.^4^ This is in agreement with our case report that the patient was affected by *Aspergillus* rhinosinusitis. Critically ill patients with severe COVID‐19 can also be susceptible to mycoses; particularly, secondary fungal infections caused by *Aspergillus* and *Candida* spp. are increasingly described.^10^, ^11^ However, *Aspergillus* co‐infection in patients with COVID‐19 pneumonia can lead to acute respiratory distress syndrome too. Aia Mohamed et al. reported 38 published COVID‐19 associated invasive pulmonary aspergillosis (CAPA) cases that most of them were caused by *A. fumigatus*.^9^ Also, Susan K. Sebastian et al. reviewed three cases of COVID‐19 associated with invasive fungal sinusitis, one of which was related to *Aspergillus*, and the rest showed zygomycosis (mucormycosis).^10^ Among patients with COVID‐19 associated fungal infections, the timely administration of antifungal therapy is paramount for a favorable outcome, particularly for aspergillosis. Consistent with the data described in this case, we need to increase our efforts to understand the full extent of *Aspergillus* infections complications in COVID‐19 and other immune disorders, and to design the best and timely diagnosis and therapy. Accurate diagnosis of the disease can be determined by the existence of consistent clinical symptoms, specific radiological aspects and mycological data.^17^ Moreover, this can reflect the fact that prophylaxis with appropriate antifungals and hospitalization of susceptible patients in isolated conditions can affect the incidence of *Aspergillus* infections in patients with COVID‐19 pneumonia.

## 4 CONCLUSION

Invasive *Aspergillus* rhinosinusitis in critically ill COVID‐19 patients affected by hematological malignancies is a condition with significant potential morbidity and mortality complications. In conjunction with paraclinical factors (imaging characteristics, histological results, and mycological techniques), the clinical manifestation can lead to an early diagnosis and a better prognosis. Moreover, antifungal prophylaxis is important for increasing survival in these patients.

## CONFLICTS OF INTEREST

The authors declare that they have no conflicts of interest.

## AUTHOR CONTRIBUTIONS

NH: contributed to this manuscript by routine laboratory examinations and writing the manuscript. RB: contributed to the patient's care; initially diagnosed the patient; and performed the follow‐up care. MA and MJN: contributed in both editing the manuscript and the required case information, images, slides. HZ: (corresponding author) contributed to this manuscript by collecting information for the case report. All authors: reviewed and approved the final manuscript.

## ETHICAL APPROVAL

We took written informed consent from the patient for publication of the case report in an anonymous way.

## CONSENT

A signed informed consent form was obtained from the accompanying patient permitting the use of the data for educational/research purposes prior to the procedure.

## Data Availability

Written informed consent was obtained from patient and is available for provision to the journal on demand.
